# An Adaptive Mechatronic Exoskeleton for Force-Controlled Finger Rehabilitation

**DOI:** 10.3389/frobt.2021.716451

**Published:** 2021-09-30

**Authors:** Thomas Dickmann, Nikolas J. Wilhelm, Claudio Glowalla, Sami Haddadin, Patrick van der Smagt, Rainer Burgkart

**Affiliations:** ^1^ Orthopaedic Research, Clinic for Orthopaedics and Sport Orthopaedics, Klinikum Rechts der Isar, Technical University of Munich, Munich, Germany; ^2^ Chair of Robotics and System Intelligence, Munich School of Robotics and Machine Intelligence, Technical University of Munich, Munich, Germany; ^3^ Machine Learning Research Lab, Volkswagen Group, Munich, Germany; ^4^ Graduate School of Systemic Neurosciences, Ludwig-Maximilians-University Munich, Munich, Germany; ^5^ Department of Artificial Intelligence, Faculty of Informatics, Eötvös Lórand University, Budapest, Hungary

**Keywords:** manipulator, interaction, exoskeletal assist system, adaptive control, rehabilitate, exoskeletal analysis, assisstive technologies

## Abstract

This paper presents a novel mechatronic exoskeleton architecture for finger rehabilitation. The system consists of an underactuated kinematic structure that enables the exoskeleton to act as an adaptive finger stimulator. The exoskeleton has sensors for motion detection and control. The proposed architecture offers three main advantages. First, the exoskeleton enables accurate quantification of subject-specific finger dynamics. The configuration of the exoskeleton can be fully reconstructed using measurements from three angular position sensors placed on the kinematic structure. In addition, the actuation force acting on the exoskeleton is recorded. Thus, the range of motion (ROM) and the force and torque trajectories of each finger joint can be determined. Second, the adaptive kinematic structure allows the patient to perform various functional tasks. The force control of the exoskeleton acts like a safeguard and limits the maximum possible joint torques during finger movement. Last, the system is compact, lightweight and does not require extensive peripherals. Due to its safety features, it is easy to use in the home. Applicability was tested in three healthy subjects.

## 1 Introduction

Patients suffering from impaired hand functionality are severely disadvantaged in performing activities of daily living (Birch et al., 2008; Wang et al., 2009; Heo et al., 2012; Cempini et al., 2015; Conti et al., 2017; Yue et al., 2017). Limited mobility of the hand can be caused by various conditions. According to Heo et al. (2012), typical examples are neuromuscular diseases, damage to the hand due to injuries, restricted motor functions as a result of a stroke, and age-related limitations.

Another condition that affects hand functionality is complex regional pain syndrome (CRPS). CRPS patients suffer from spontaneous, deep-seated pain and are often severely hypersensitive to stimuli or touch. In addition, almost all patients have motor weakness and marked limitation of movements such as fist closure and pincer grip (Maihö fner et al., 2010). Conventional therapy is time-consuming and often leads to unsatisfactory results because patients are discharged from the hospital too early, therapists are not available in sufficient numbers, and the overall financial burden is high (Epstein et al., 2008; Elsamadicy et al., 2017). For this reason, there are efforts to restore or at least improve hand functionality through the use of robotic rehabilitation systems. Important findings from research on stroke rehabilitation in the context of robotic hand rehabilitation were highlighted by Heo et al. (2012), emphasizing their potential in therapy: Reinkensmeyer et al. (2004) have shown that recovery from brain injury is strongly influenced by the patients’ sensorimotor experiences after the injury. The supportive role of repetitive motion training in the recovery of motor skills has been shown by both Taub et al. (1993), Mark and Taub (2004). Furthermore, according to Patton and Mussa-Ivaldi (2004), hand rehabilitation using repetitive motion training with a robotic system is most likely more effective than conventional therapy.

In contrast to manual therapy, exoskeletons equipped with suitable sensors can provide accurate and repeatable data to quantify the functional status of the patients hands. Additionally, high-frequency rehabilitation exercises do not increase the financial burden of the treatment. Therefore, using exoskeletons in therapy can allow patients to follow a daily training routine, which is usually not possible in manual therapy. Although several exoskeleton systems have been developed and their potential for rehabilitation has been demonstrated, the use of exoskeleton systems in therapy is still limited. Yue et al. (2017) note that only a small subset of the exoskeleton systems developed make it into clinical testing or practice due to their complexity and resulting poor usability in a clinical context. In addition, the development of a hand exoskeleton presents many technical challenges due to the limited space available and the complex biomechanics of the human hand with its many degrees of freedom.

A kinematic structure that is, both simple and scalable for therapy use was developed by Jo and Bae (2017), likely as an extension to an approach by Festo AG and Co. KG (2012). Because it combines a single electric motor to actuate each finger with adaptive kinematics, this system can scale to the entire hand. However, the full potential of the kinematic approach is not realized, as the trajectories of the finger joints and the associated forces in the joint are not captured.

In this work, we extend the approaches of Festo AG and Co. KG (2012), Jo and Bae (2017) by adding position sensing, a more user-friendly bi-directional actuator and force sensor. We also provide a mathematical model to represent the actuation forces on the load in the finger joints.

## 2 State of the Art Wearable Hand Interaction Systems

Over the past 2 decades, a variety of hand exoskeleton systems have been developed, most of which are intended either for hand rehabilitation, force assistance, or to provide haptic feedback, e.g., for virtual-reality applications.

**FIGURE 1:**
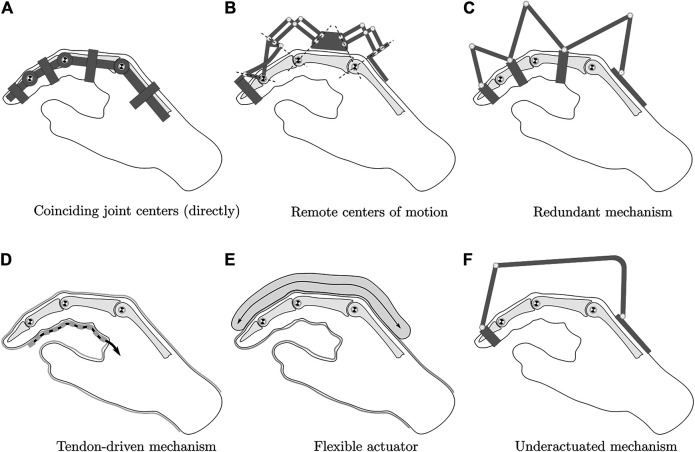
Hand exoskeleton kinematic options, modified from Heo et al. (2012).

Regardless of the intended use, all systems must meet the key requirement of biomechanical compatibility with the human hand to enable safe operation. As described in Heo et al. (2012), the exoskeleton is functionally coupled to the hand when worn. The exoskeleton must not impose any movement that disregards the natural pivot points of the finger joints. To ensure this, several kinematic concepts can be used, as shown in Figure 1. The exoskeleton can be built to directly align with the pivot points of the finger joints (concepts A and B in Figure 1). However, different phalanx lengths between users lead to misalignment of the joint centers. The remaining four concepts solve this problem by not imposing a defined center of rotation on the finger joints. This can be achieved by incorporating the skeletal structure of the finger into the kinematics. With redundant actuation of the joints, each joint is incorporated into a four-bar linkage with one degree of freedom (C), which can then be used to actuate each joint independently. Alternatively, flexible actuation systems such as tendons (D) or artificial muscles (E) without inherent stiffness can be used, as well as a rigid but underactuated system (F).

For the given application, direct matching of the joint centers is not a viable option, since the system is intended to be used on a variety of different patients. A flexible kinematic structure is also disregarded as an option, as a rigid kinematic structure is required to precisely recreate the finger joint configuration from positional sensor data obtained at the kinematic structure. Depending on the chosen kinematic concept, the transmission of the actuation force to the individual finger phalanges represents a key challenge. In principle, two actuation concepts can be distinguished: Direct and indirect drive. Many systems deploy indirect actuation systems using Bowden cables because of their ability to transmit actuation forces to multiple finger phalanges and in various finger joint configurations. However, as the review of these systems shows, indirect actuation compromises accuracy due to friction and backlash, as discussed in Wege et al. (2006), Chiri et al. (2009), Iqbal et al. (2014). To obtain precise actuation force data, direct actuation is therefore preferred.

In the following, exemplary systems are presented which use redundant or underactuated kinematic concepts. The system developed by Wege et al. (2006) has four active degrees of freedom. It is capable of actuating all joints of all fingers *via* Bowden cables, including abduction and adduction of the MCP joints. Kinematically, the system deploys a redundant coupling mechanism to ensure biomechanical compatibility with the hand, corresponding to concept C in Figure 1. Their experiments with the system show that both force and position control are problematic due to the internal friction of the Bowden cables and the resulting disturbances and nonlinearities in the controlled system.

A simpler system with direct actuation via a linkage mechanism was developed by Iqbal et al. (2014). One linkage mechanism moves the index finger and a second one moves the thumb. Both mechanisms are individually actuated by a single motor, resulting in an underactuated system corresponding to concept F in Figure 1. The system provides a total of 4 degrees of freedom, however, only flexion, and extension of the MCP and PIP joint can actively be actuated by applying force to the middle phalanx. The motors provide force control of the setup. However, due to the nature of the simple mechanism, it is impossible to calculate the individual joint forces or angles of the finger. When the linkage mechanism is attached to the proximal phalanx of the index finger, force and position control of the MCP joint becomes possible by incorporating the joint into a single four-bar mechanism. However, the PIP and DIP joints then are no longer actuated and only follow finger movement passively.

Chiri et al. (2009) developed HANDEXOS, a system for stroke rehabilitation. It uses a kinematic structure with direct matching of the joint pivot points corresponding to concept A in Figure 1. However, it deploys compliant finger attachments to compensate for variations in the phanalx lengths of various patients. The underactuated system is able to actuate all finger joints in terms of flexion and extension, it additionally follows passively the abduction and adduction of the MCP joint. The system therefore provides three active and one passive degree of freedom. By modifying the actuation unit, it also allows independent actuation of each joint. It uses Bowden cables to transmit the actuation force to the finger joints and is thus subject to the same force control challenges as the Wege et al. (2006) system.

The system developed by Leonardis et al. (2015) adaptively actuates all long fingers with a single motor and is characterized by its electromyographically (EMG) controlled concept, which transfers the measured gripping force on the healthy hand to the pathological hand. The gripping force is measured directly via force sensors on the objects to be gripped. The kinematics are a modified variant of concept C in Figure 1, in which the actuation of all three joints are coupled *via* additional links.

AMADEO by Tyromotion (2019) is a commercially available rehabilitation system. The fingers are connected to linear actuators using magnets, which are taped to the patient’s fingertips. The system features EMG sensors to read the patient’s intended motion and offers multiple rehabilitation modes. All five fingers can be moved by the system and diagnostic data can be recorded during the exercises, including finger force and range of motion. However, due to the underactuated concept with a single point of contact, no information can be obtained on the joint level.

Conti et al. (2017) present a model-based approach to create a cable-driven, one degree of freedom exoskeleton mechanism which is optimized to follow the phalanx trajectories of a specific patient’s hand. The trajectory of the patient’s index finger phalanges is captured visually, but no force sensor data is recorded by the exoskeleton system itself. The kinematic structure can be interpreted as a variant of concept C in Figure 1 with the finger joints being incorporated in mechanisms with a single degree of freedom each. However, instead of using three four-bar mechanisms with rotational joints, a kinematic chain with rotational and translational joints is deployed.

A system with a force sensor concept was developed by Aragón-Martínez et al. (2020) for teleoperation purposes and actuates two fingers in one housing. However, the system cannot distinguish between the individual finger forces and the fingers are only guided *via* the fingertip. The kinematic structure uses remote center of motion mechanisms and corresponds to concept B in Figure 1. The exoskeleton features an FSR-style force sensor to read the force-feedback at the fingertip.

The system developed by Lee et al. (2018) is capable of independently actuating the MCP and PIP joints of the fingers and therefore provides two active degrees of freedom. A rigid kinematic structure comprised of two coupled linkages, a four-bar and a six-bar linkage, allows direct transmission of the actuation force to the proximal and medial finger phalanges. The kinematic concept is a variant of concept C in Figure 1. The contact forces to the finger are measured using load cells. However, the DIP joint is not included in the movement and the need for independent joint control makes the system less flexible for training purposes in comparison to an adaptive system.

An adaptive concept was developed by Sarac et al. (2016) that optimizes the direction of force application and controls the MCP and PIP joint *via* an actuator. The concept features orthogonal force application and is optimized for a large range of motion and different hand sizes and was extended by Di Guardo et al. (2019) to include an adaptive calibration algorithm for improved kinematic parameter adjustment.

An alternative concept to the highly adaptive systems with only one motor is the approach of Carbone et al. (2020). By means of optical tracking of the movement space of the fingers, a system was created that can actively control two degrees of freedom and represents a good compromise between freedom of movement and control. The approach by Jo and Bae (2017) uses an underactuated kinematic structure with three degrees of freedom that incorporates all three finger phalanges. The kinematic concept is identical to the Festo ExoHand (Festo AG and Co. KG, 2012), which was presented in 2012. This kinematic structure allows the computation of all forces transmitted to the finger phalanges, provided that the configuration of the mechanism and the actuator force acting on the structure are known. Thus, unlike underactuated systems with a single point of contact to the finger, the individual joint moments of all finger joints can be calculated. The Jo and Bae (2017) system is intended for VR applications and features monodirectional force control using a series elastic actuator (SEA) mechanism. With this actuation concept, the exoskeleton is only capable of transmitting resistive forces to the fingertips and is therefore unsuitable for rehabilitation. In contrast, the ExoHand is capable of applying bidirectional forces and can be used to assist movement with precise force and position control of the actuators. However, the pneumatic drive system and the required peripherals make it difficult to use the system in clinics or private homes. In addition, because the ExoHand is augmented with additional artificial phalanges that require precise joint positioning, the system must be customized for each user.

Neither system has position sensors to fully replicate the kinematic configuration. Only the actuator position is captured by a sensor to realize position control of the actuator. However, due to the adaptivity of the system, this is not sufficient to allow the calculation of finger joint angle and torque trajectories. In general, the kinematic structure of Jo and Bae (2017), Festo AG and Co. KG (2012) holds great potential for rehabilitation purposes as it has three degrees of freedom and allows natural and unrestricted finger movement.

To fully exploit this potential, we extend the approaches of Festo AG and Co. KG (2012), Jo and Bae (2017) by adding angular position sensing to the kinematic structure and a bi-directional electric linear actuator coupled to a force sensor. By measuring the angles between linkages at three joints of the kinematic structure, a parameterized dynamic model of the index finger can be obtained. Thus, both tracking of joint motion for determining patient progress, as well as accurate mapping of measured forces and resultant loads/resistance torques along each joint becomes possible. Additionally, individual joint angle or torque responses can be used as an input for the actuator control system.

## 3 Exoskeleton Platform

### 3.1 System Requirements

To ensure success in the practical application of the rehabilitation system, a number of requirements must be met: First and foremost, the system must be safe and not hinder the patient. In order to achieve this, the exoskeleton must be biomechanically compatible with the human hand as discussed above. Hyperextension of the finger joints and the application of critical force levels by the exoskeleton must be avoided as well.

Since the exoskeleton is to be used for rehabilitation exercises consisting of various grasping tasks, the resulting movement must be designed for a large working range and without constraining forces. Hand sizes vary, so the system design should account for such variations.

To allow the patient to interact with objects during training sessions, the palm should remain free. All three finger-flexion joints are supposed to be stimulated during the movement and diagnostic data should be recorded in the form of angle and torque trajectories for each joint.

The kinematic concept should be scalable so that the system can be extended to all fingers of the hand. The overall complexity of the system must be low enough to be suitable for clinical and home use. Finally, the system should be comfortable to wear and easy to attach and detach from the patient’s hand.

The hardware/architecture of the exoskeleton is supposed to allow multiple rehabilitation modes, namely active, active-assistive, and resistive training.

Considering these requirements, a number of necessary features can be identified. To prevent hyperextension of the finger joints, even if the control system behaves incorrectly, Heo et al. (2012) propose to deploy mechanical end stops. Furthermore, the force control must allow fine adjustment of the maximum actuation forces acting on the fingers in order to calibrate the training intensity according to the current functional state of the patient’s hand. The kinematic structure must be rigid to allow discrete and precise positioning and accurate interpretation of the read sensor data in the kinematic model. To ensure natural finger motion, an underactuated system with three degrees of freedom is preferred over a system with individually actuated joints. The different rehabilitation modes require bidirectional force support with up to approximately 45 N actuation force [Iqbal et al. (2009); Iqbal and Khelifa (2014)]. In order to provide precise force control, direct control of the kinematic structure is required. The requirement for low complexity and good mobility of the system argues against pneumatic control.

We summarize the requirements as follows:

1) Safety of use;
2) Suitable for clinical and home use;
3) Scalability for use on the whole hand;
4) Easy to wear;
5) Optimized for a large range of motion;
6) Movement freedom of the palm;
7) Angle and torque measurement for each joint;
8) Compatible for multiple rehabilitation modes.

Despite an extensive literature search, no system could be identified that sufficiently addressed all the listed requirements. However, the approach presented by Festo AG and Co. KG (2012) and later modified and iterated by Jo and Bae (2017) came close, which made us choose it as the direct basis for the common structure presented in this paper.

### 3.2 Kinematic Model

**FIGURE 2:**
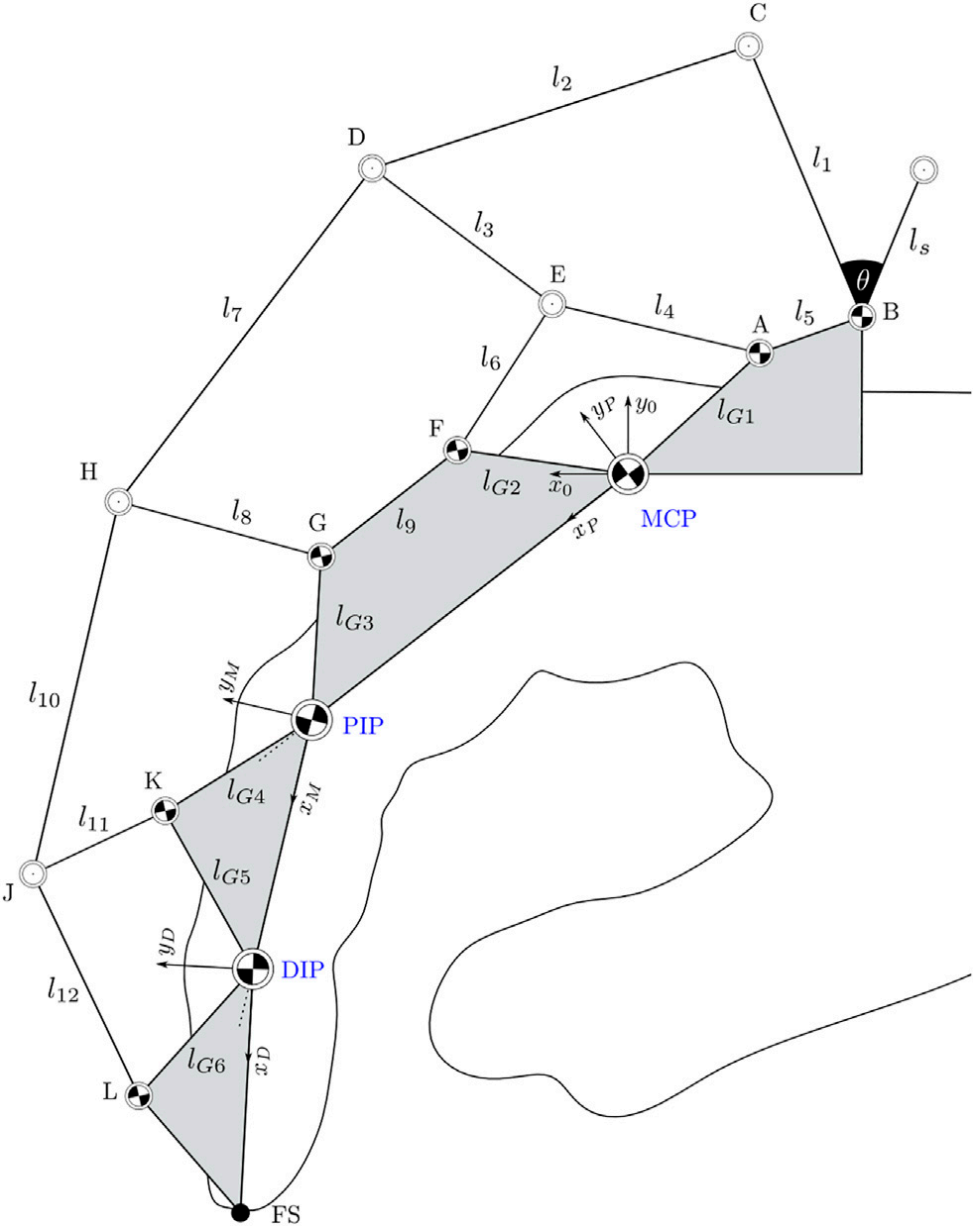
Linkage structure of the exoskeleton system as an extension to Jo and Bae (2017). The finger joints are marked in blue. Areas marked in grey are considered as rigid bodies within the structure.

The kinematic structure of our approach is visualised in Figure 2. The rigid links *l*_1_, *l*_2_, *l*_3_, *l*_4_, *l*_6_, *l*_7_, *l*_8_, *l*_10_, *l*_11_, and *l*_12_ lie in the plane of the flexion/extension motion of the finger. Their axes or rotational axes are parallel to the axes of the finger joint and they are connected by eleven rotational joints (A–L) with six points of connection to the finger and the back of the hand (A, B, F, G, K, L). The entire kinematic chain is composed of the combination of the rigid links and the connecting rotational joints, as well as the skeletal structure of the finger and the MCP, PIP, and DIP joints. The kinematic chain can be decomposed into three five-bar linkages, each with two degrees of freedom, and two four-bar linkages, each with one degree of freedom. This structure allows for greater flexibility and ROM compared to simpler variants with fewer subsets of joints, as shown in Jo and Bae (2017). In addition, incorporating the MCP joint into a four-bar linkage results in the ability to control flexion and extension of that joint individually, as was done for index finger kinematics in Festo AG and Co. KG (2012).

In order to determine the forward kinematic of the exoskeleton the degrees of freedom need to be determined. The total number of degrees of freedom for a planar mechanism can be calculated according to Kerle et al. (2015) using Eq. 2 with *n* = 14 representing the numbers of links in the mechanism, and *g* representing the number of joints. Joints connecting three links instead of two are counted twice. This yields 10 + 2 ⋅ 4 = 18 joints. Finally, *f*_*i*_ represents the degrees of freedom for each joints, which in this case is 1 for every counted rotational joint.

$$\begin{array}{ll} F & =3\;\left(n-1\right)-3g+\overset{g}{\underset{i=1}{\sum }}f_{i} \end{array}$$ (1)

$$\begin{array}{ll} & =3\;\left(14-1\right)-3\cdot 18+18=3 \end{array}$$ (2)

The three degrees of freedom of the exoskeleton obtained by the calculation match the degrees of freedom of the finger joints. Consequently, at least three relative angles between the joints must be known to fully reconstruct the configuration.

The kinematic model can be efficiently calculated using the tan-half-angle technique presented in McCarthy and Soh (2011), p. 412. Using this method, the two potential positions of a joint can be calculated based on its respective distance relative to two known joints. Geometrically, these solutions represent the two intersections of circles with known diameters around the two known joints. This geometric constellation of three points in space will be referred to as the “two-stroke” in the following. Successive application of the tan-half angle method efficiently yields both a forward and a backward kinematic model. Suppose a point *P* with unknown coordinates has distance *d*_1_ to a known point *M* and distance *d*_2_ to a known point *N*.

First, the parameters *X*, *Y*, and *Z*_*X*_ are calculated:

$$X=N_{x}-M_{x}$$ (3a)

$$Y=N_{y}-M_{y}$$ (3b)

$$Z_{X}=\frac{X^{2}+Y^{2}+d_{1}^{2}-d_{2}^{2}}{2\;d_{1}}$$ (3c)

**FIGURE 3:**
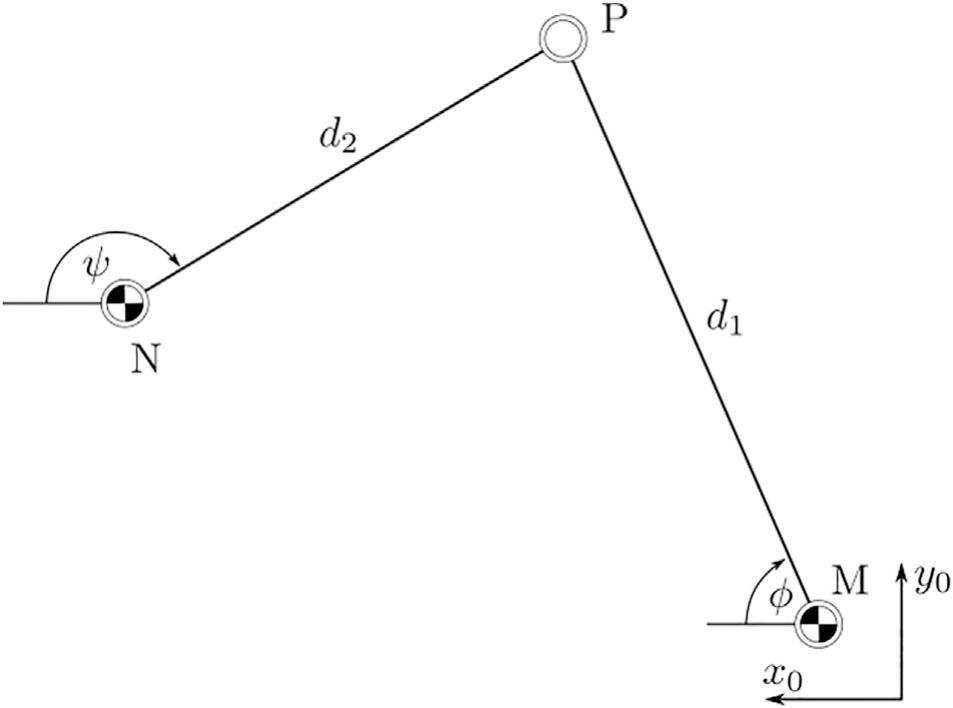
Orientation of points P, M, and N relative to a world frame as defined in Figure 2.

Using these parameters, the angle between the *x*-axis of the reference frame *x*_0_, *y*_0_ and the vector from point *M* to point *P* can be calculated (see Figure 3):

$$\phi =2\cdot \arctan\left(Y\pm \sqrt{\frac{X^{2}+Y^{2}-Z_{X}^{2}}{X+Z_{X}}}\right)$$ (4)

The solution must be defined by explicitly setting the sign in Eq. 4. For *M*_*x*_ < *N*_*x*_, the positive solution in Eq. 4 provides the upper candidate solution, i.e., the candidate solution for the point *P* with the higher value of *P*_*y*_ in the reference frame. Finally, the coordinates of the point *P* are given by:

$$P_{x}=M_{x}+\cos\phi \cdot d_{1}$$ (5a)

$$P_{y}=M_{y}+\sin\phi \cdot d_{1}$$ (5b)

A direct kinematic model is required for the application. Based on the measured angles *ϕ*_A_ in joint A (link *l*_4_ relative to *x*_0_), *ϕ*_B_ in joint B (link *l*_1_ relative to *x*_0_) and *ϕ*_K_ in joint K (link *l*_11_ relative to *x*_*M*_), the configuration of the kinematic structure is calculated. The location of the joints A, B, and MCP are initially known. The angles *ϕ*_A_ and *ϕ*_B_ yield the position of the joints C and E. Using these positions, applying the presented two-stroke concept to the joint trio E, C, and D then yields the location of joint D, completing the first five-bar linkage of the kinematic structure. Next, the position of joint F is calculated using a two-stroke applied to the joint trio MCP-E-F. As the grey links in Figure 2 represent rigid bodies, the position of joint F then yields the positions of joints G and PIP. With these known, the two-stroke of joints G, D, and H yields the position of joint H and so forth.

**FIGURE 4:**
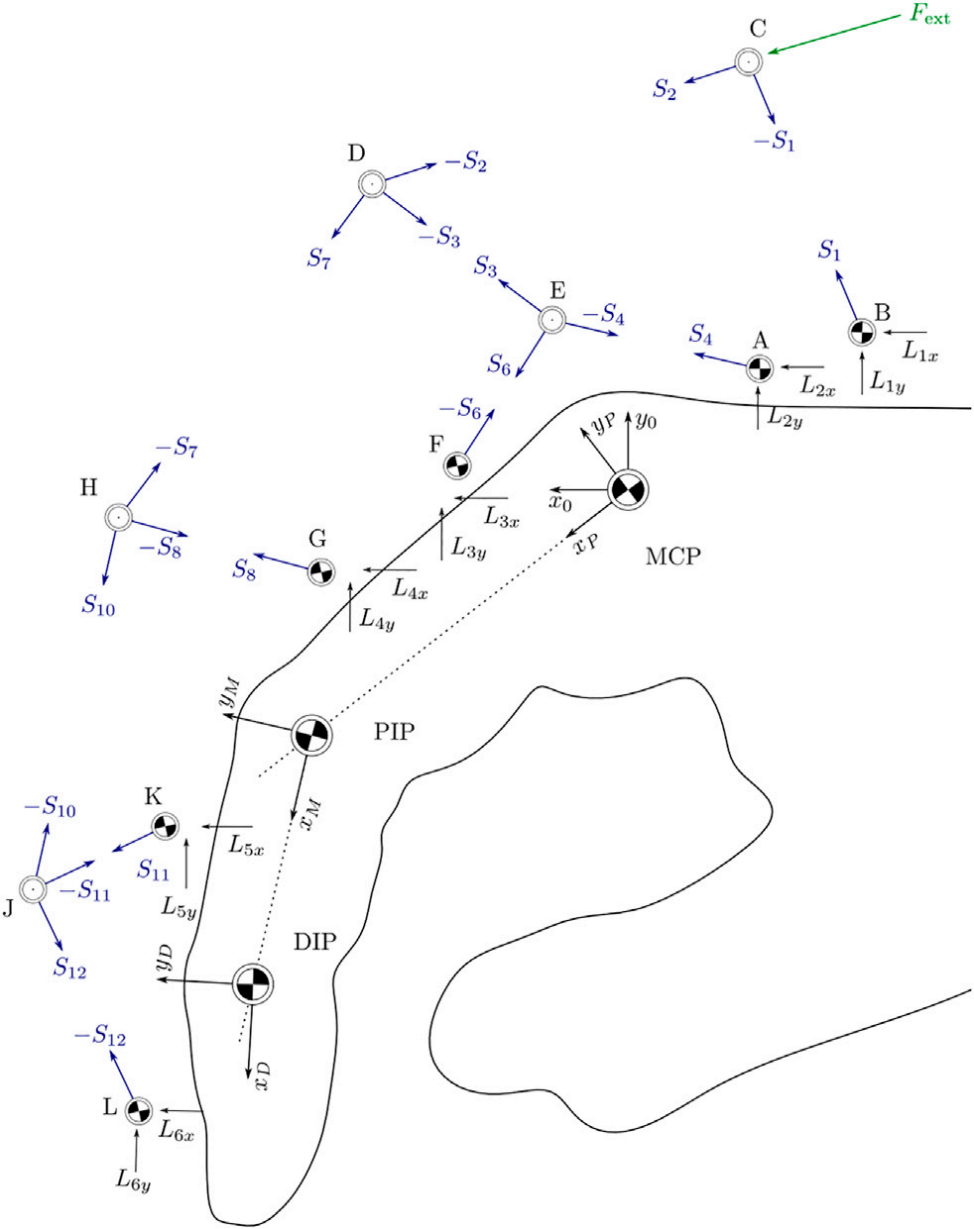
Orientation of all acting forces in space as the basis for equilibrium equations for each joint.

Once all joint positions are known, the angle of each link in the kinematic structure can be calculated with respect to the *x*-axis of the reference frame *x*_0_, *y*_0_. Since the links can be modeled as a rods, transmitting force exclusively along their respective center lines, the orientation of all forces acting in the kinematic structure is thus known, as seen in Figure 4. Decomposition of each force into its components along the *x*_0_- and *y*_0_-axis then allows the derivation of 22 equations of force equilibrium around the 11 joints A–L. This linear system of equations is fully defined and can then be solved for the 10 unknown forces along the links and the 12 unknown contact forces in the joints A, B, F, G, K, and L represented in the reference frame. Using this solution, the contact forces on the finger phalanges can be directly calculated from any known external force applied by the actuator at the joint C. The detailed calculations are included in the GitHub repository.

**FIGURE 5:**
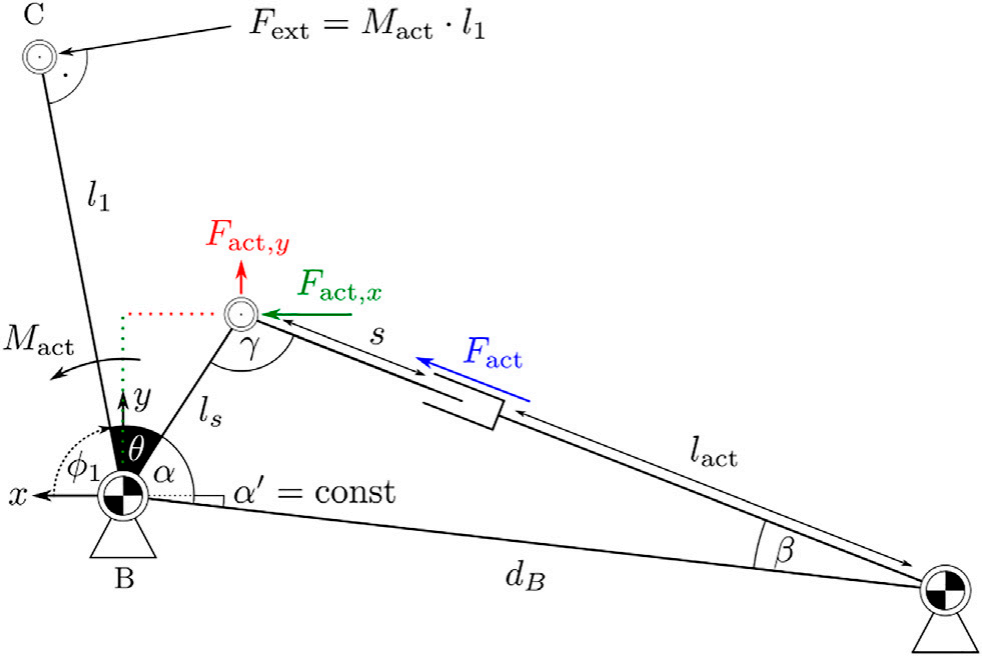
Kinematic connection between actor and exoskeleton. The force *F*_act_ generates the external momentum *M*_act_ around point B.

The system uses a linear actuator, as shown in Figure 5. In this way, multiple actuators can be placed on the dorsal side of the hand so that all fingers can be actuated simultaneously. The angle *ϕ*_1_ is known from the kinematic calculations. By additionally measuring the actuation force *F*_act_, the external force *F*_ext_ acting on the kinematic structure can be determined.

### 3.3 Dynamic Model

**FIGURE 6:**
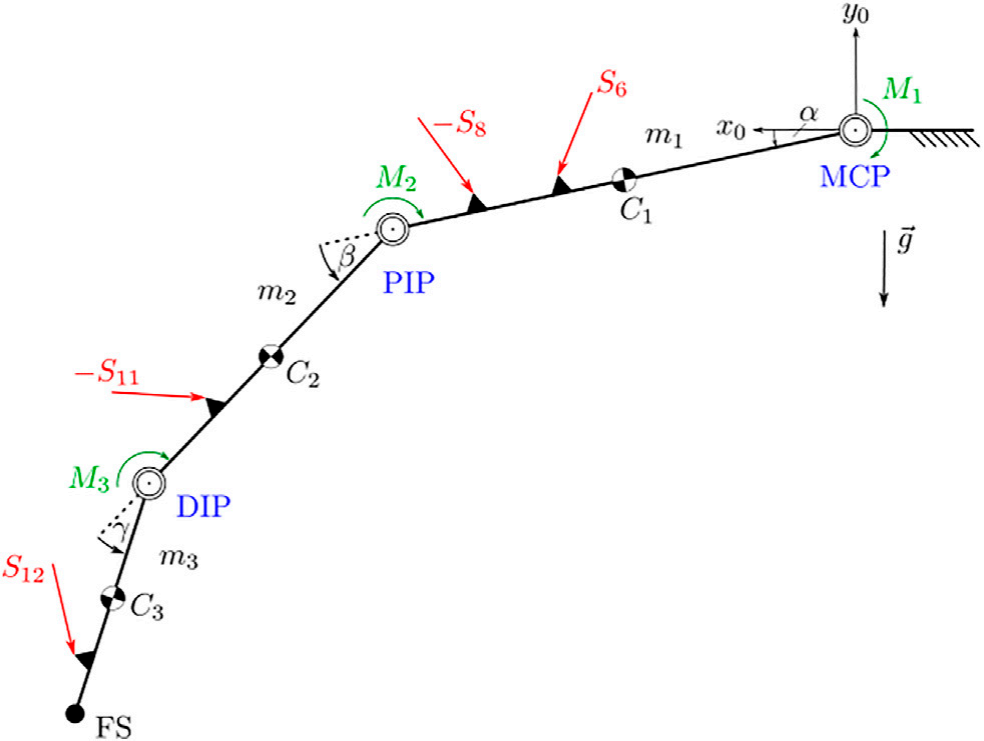
Dynamic model of the unconstrained finger. The finger joints are marked in blue. Areas marked in black are considered as rigid bodies within the structure. External forces are marked in red.

With the use of the contact forces between the exoskeleton and finger, a dynamic model, as shown in Figure 6, can be derived. The model consists of the three finger joints moving in a plane and connected by revolute joints. An unknown torque (*M*_1_ − *M*_3_) acts in each joint, representing the joint resistance to motion or an actuation torque applied by the patient. The minimum coordinates ***q*** = (*α*,*β*,*γ*)^*T*^ of the dynamic model and the contact forces are known from the previous calculations. The dynamic model can be solved for the unknown torques *M*_1_ − *M*_3_ in the case of unrestrained motion, i.e., no additional contact forces act on the finger using the projected Newton-Euler equations:

∑i=13JT,iTIFsum+JR,iTKiMsum=0,withFsum=miaCiI−FiI,andMsum=L˙iKi+ωiKi×LiKi−MiKi. (6)

For each body in the system, the sum of forces and moments is calculated and projected in the directions compatible with the kinematic constraints via the transposed individual Jacobian matrices for translation JT,iTI and rotation JR,iTKi . The forces *F*_*i*_ acting on each body are described with respect to the inertial reference frame *I* while the moments *M*_*i*_ are described in body-fixed coordinate frames *K*_*i*_. *L*_*i*_ represents the angular momentum and *ω* the angular velocity of each body.

Since the moving masses in the system and their velocities and accelerations are small compared to the external forces and moments acting on the system, the model can be approximately described quasi-statically with $\dot{q}=\ddot{q}=0$ . This allows a sufficiently accurate calculation of *M*_1_ − *M*_3_ based on the data from the kinematic calculations alone. If required, the kinematic calculation can be extended by $\dot{q}$ and $\ddot{q}$ for the dynamic case.

## 4 System Integration

**FIGURE 7:**
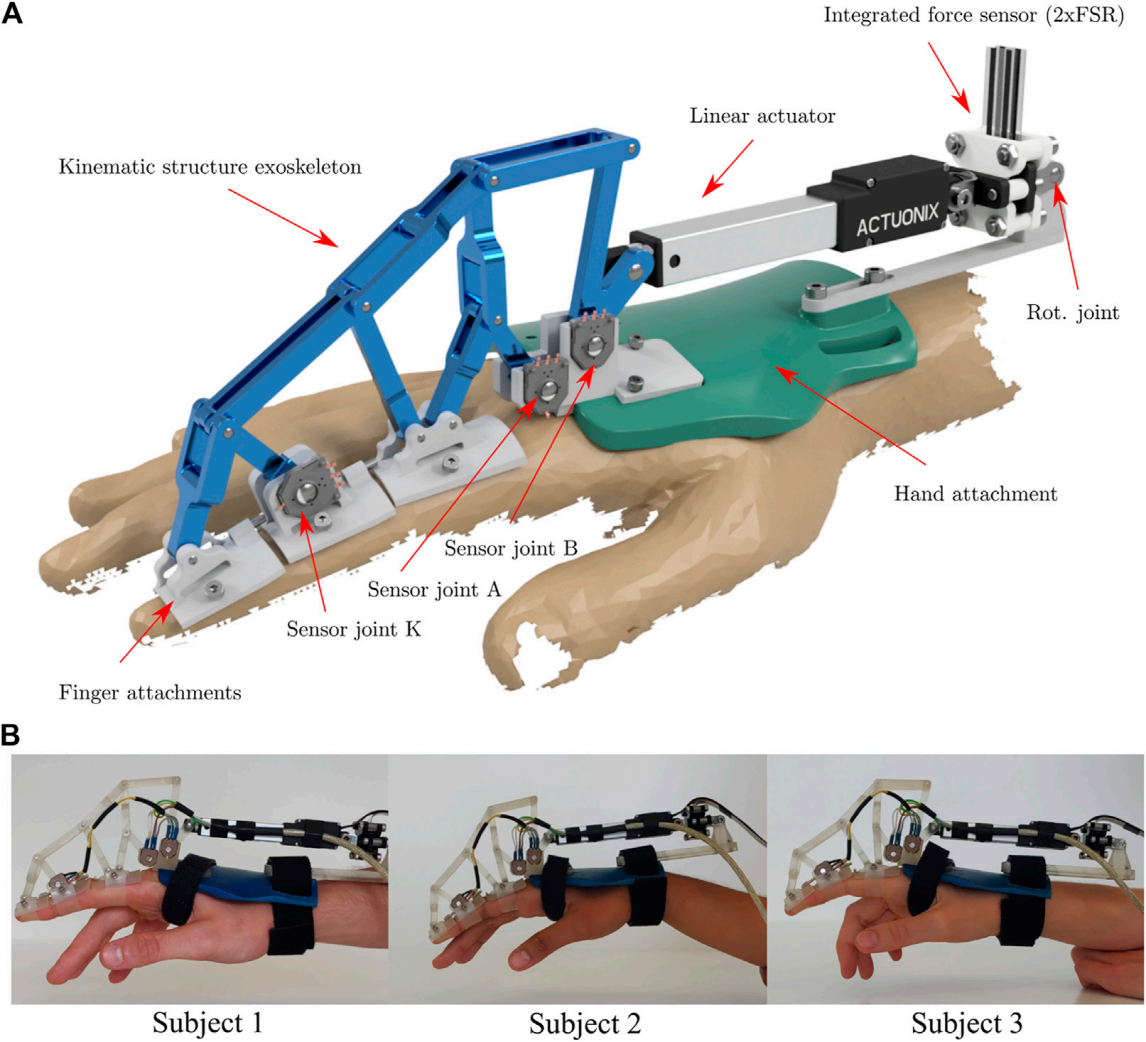
Rendering **(A)** and application of the real system **(B)** of the exoskeleton. The rendering contains all the information about the exoskeleton and its components. In the bottom row, the exoskeleton is attached to the index finger of three individual test subjects.

To test the functionality, adaptability and limitations of the system, a prototype was built and tested on three individual subjects. The prototype and its use on the three test subjects is shown in Figure 7. To ensure sufficient rigidity of the system, the flange between the motor mount and the back of the hand was reinforced, as shown in the bottom row of the figure.

Joints A and B were selected for potentiometer placement because they have a large range of motion that allows good utilization of sensor resolution, and because they are well suited for mechanical reasons: They are not displaced during gripping/finger movements, which reduces the complexity of cable management, and their location on the dorsal side of the hand facilitates sensor mounting. Joint K was chosen because it also has a satisfactorily large range of motion and the finger attachment offers more flexibility in designing a sensor attachment than the kinematic joints.

The finger attachments were realized with silicone bands that are variably adjustable by means of a clamp connection with a screw. This ensures sufficient force transmission to the respective finger limb. To avoid hyperextension of the finger joints, the attachments are extended on their proximal side with small screws to connect to the next attachment before hyperextension can occur. In addition, the connection between the motor and the exoskeleton kinematics is realized by a small pin that allows a quick and safe release of the motor if necessary. For electrical safety, the 12 V power supply of the linear motor is coupled with an emergency stop switch that can be used to switch off the motor at any time.

**FIGURE 8:**
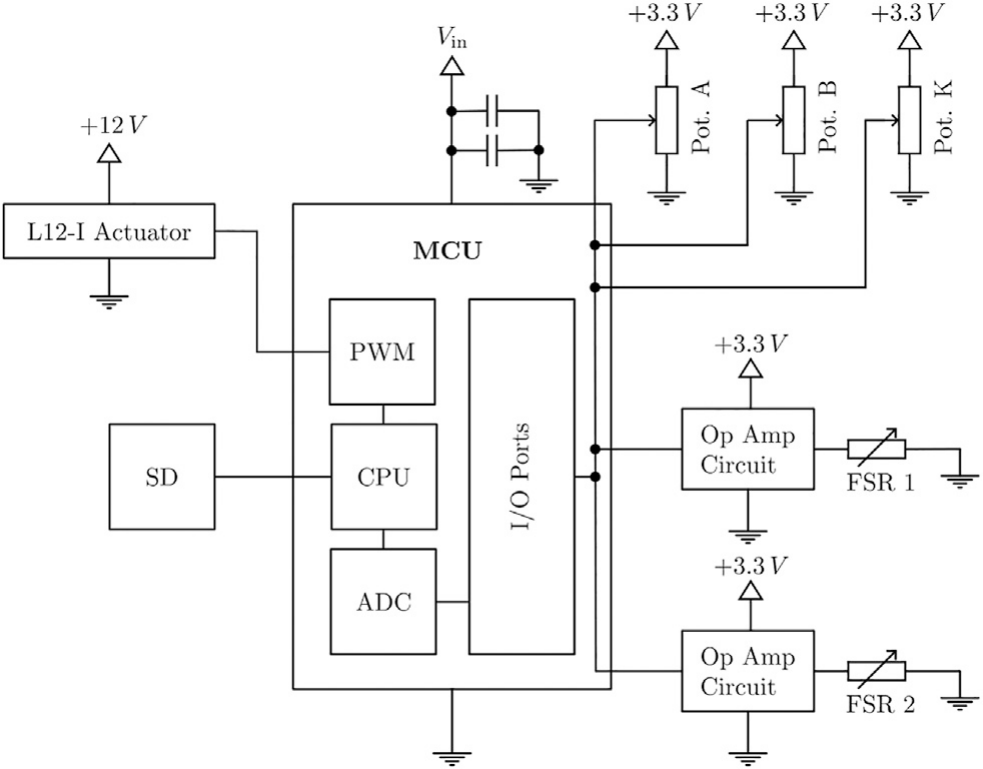
Interconnection of all electric components of the exoskeleton system.

Figure 8 displays the architecture of the sensor control system. Three rotary potentiometers were used as angular position sensors. To increase the accuracy of the position sensors and to compensate for the non-linear behaviour of the potentiometers, discrete ADC counts were determined at 2°degree intervals. Linear interpolation between these calibration points resulted in sufficient position accuracy.

The position and force sensor readings are obtained by an ADC on a Teensy 4.1 microcontroller with a 12-bit resolution using a moving average filter to smooth the signal. The raw ADC values are logged onto an SD card with a frequency of 100 Hz. Additionally, the momentary actuation force is calculated in real time on the microcontroller as an input for the control system. Based on the output of the control system, a L12-I actuator by Actuonix® is positioned *via* a PWM signal.

### 4.1 Force Sensor

**FIGURE 9:**
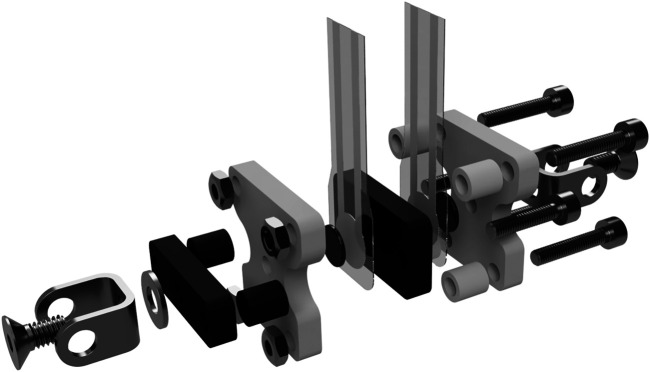
Exploded view of the force sensing resistor (FSR) sensor integration. The setup ensures an orthogonal application of the applied forces on the resistor in tension and compression direction.

To measure the actuation force, FSR-type force sensors were integrated into a custom-built mechanical enclosure shown in Figure 9. This setup allows bidirectional force measurement with satisfactory accuracy using two sensors. While strain gauge-based force sensors are superior to FSRs in terms of accuracy, the required integration effort, high sensitivity to vibration and handling, as well as their price point make them less suitable for the present application. In comparison, FSR-style sensors are highly affordable, easy to integrate, and relatively robust. To maximise the FSRs’ inherently lower performance, typical sources of errors were eliminated through the design and placement of the force sensors’ enclosure. During operation, the actuator force acts perpendicularly to both sensor surfaces at all times, while the flat and parallel surfaces of the enclosure ensure an even load distribution on the sensing surface. Many systems deploy FSR sensors at the contact points between the finger phalanges and the exoskeleton to measure the individual contact forces. In this configuration, the sensors are often subject to deformation and shear loads, as the attack angle of the external forces acting on the finger phalanges change depending on the exoskeleton pose. This substantially compromises the accuracy of the measurements. Wege et al. (2006) for example, deployed FSR sensors at the top and bottom of each phalanx, but due to increased inaccuracies were only able to measure dynamic changes in the applied forces using this setup. Using the exoskeleton kinematics and force sensor design proposed in this paper allows to obtain all contact forces at the phalanges using only two sensors per finger instead of six, while guaranteeing a constant ideal load configuration. The force sensor unit pivots freely around its rear attachment point. It is rigidly clamped to the linear actuator at its front attachment point, acting as an extension of its center line. The enclosure is made up of two rigid claws, a black and a white one as shown in Figure 9. These claws are axially unconstrained to each other, allowing minimal movement in the direction of the acuator’s center line. One FSR sensor is attached to either side of the black claw’s rear panel, which sits in between the two panels of the white claw. The rear attachment point of the sensor transmits force into the while claw, while the front attachment point (connected to the linear actuator) transmits force into the black claw. This configuration causes the actuator force to flow across the rear-side FSR upon compression and the front-side FSR upon stretching of the sensor claws.

Two FlexiForce® A201 sensors with a maximum load rating of 45 N were used in the prototype, providing measurements with satisfactory accuracy of the desired maximum force range of approximately 0–40 N. To achieve linear sensor response and to adjust the force ranges, two non-inverting operational amplifier circuits, as suggested by the manufacturer, were used to process the voltage readings from the sensors.

### 4.2 Control System

**FIGURE 10:**
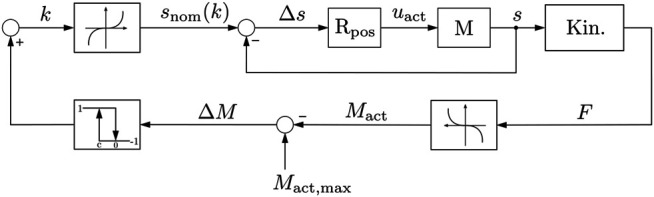
Control loop of the exoskeleton. The controller follows a predefined position signal in the inner control loop and is limited by a torque-based two-point control.

For the initial tests of the system, a passive force control system was implemented as shown in Figure 10. A desired actuator trajectory is calculated on the controller in real time and decomposed into a series of desired actuator positions *k*. These are sent to the controller and converted by its built-in position controller. The resulting actuator position *s* displaces the *l*_1_ link of the kinematic structure. The reaction force is measured by the force sensor and the corresponding actuation torque in the joint *B* is calculated. This actuation torque is compared with a threshold value and the delta is processed by a two-point controller. When the upper threshold of the controller is reached, it reverses the direction of the command actuator trajectory. As soon as the actuating torque has fallen below the lower threshold of the controller, the target trajectory is inverted again. This prevents the system from oscillating. Due to the adaptivity of the system, it is not possible to control the individual joint trajectories by manipulating the actuator position. However, the kinematic and dynamic model described above allows for a response to the system response at the joint level. When fully implemented on the MCU and interpreted for each iteration of the control loop, these models yield the joint angles and joint torques for each joint as potential input to the control system.

## 5 Experiments

To gather information about the performance of the introduced exoskeleton architectures, different experiments were conducted. Their design concentrated on the following aims:

• Accuracy of the developed force sensor unit;
• Total ROM determination of the exoskeleton for different hand sizes;
• Behavior of joint angle and torque tracking for different hand sizes;
• Verification of force-control interaction.

In order to validate the exoskeleton for different hand sizes tests were performed on three different healthy subjects.

### 5.1 Force Sensor Validation Tests

**FIGURE 11:**
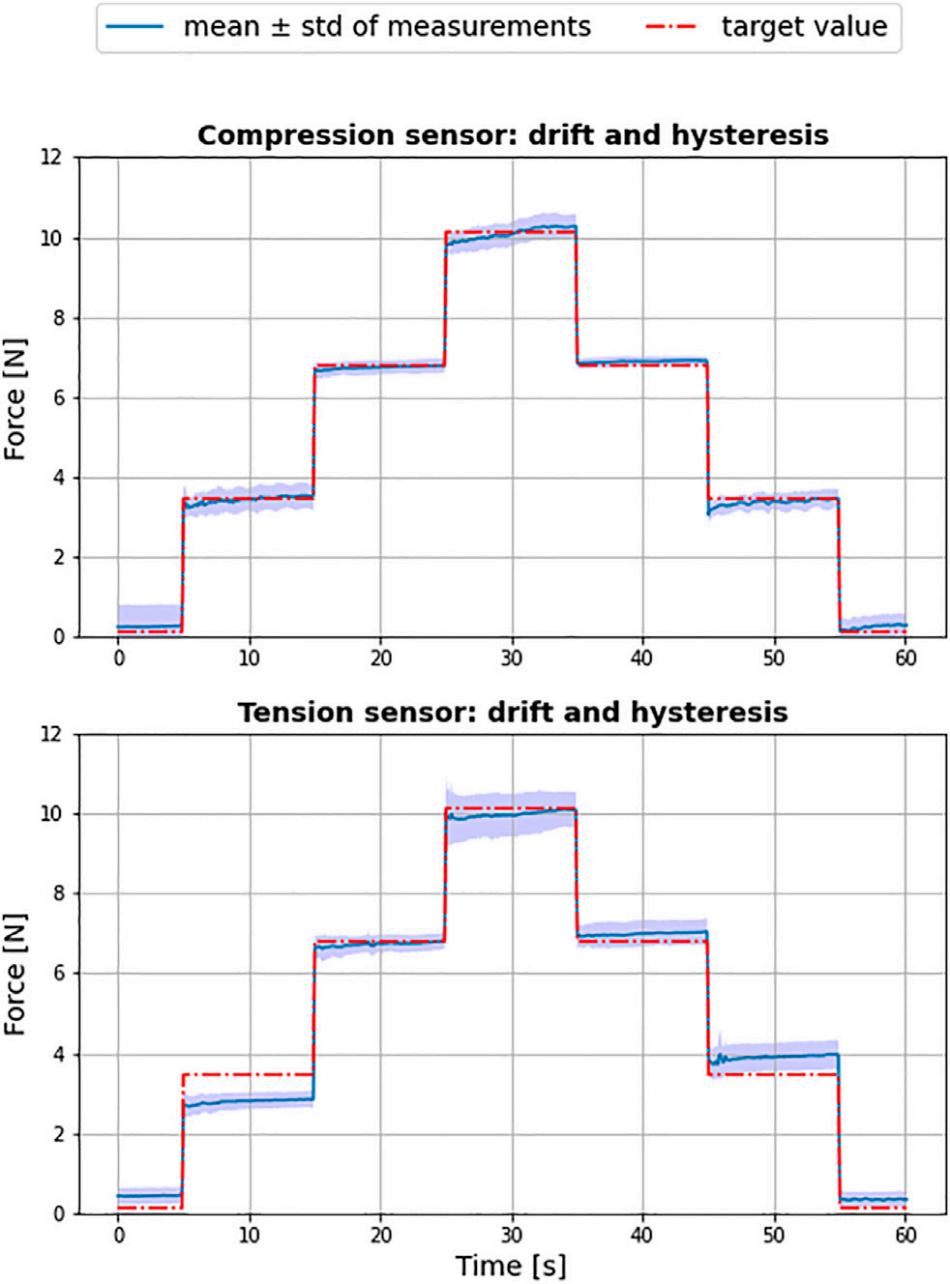
Result of force sensor tests for tension (sensor 1) and compression (sensor 2) over ten independent test cycles.

To quantify the error of the FSR sensors, experiments were conducted to analyse the sensor drift as well as the hysteresis error. Figure 11 shows the results of ten consecutive runs. The red dotted line represents the actual sensor load, the solid blue line the mean of all obtained measurements and the transparent blue band the standard deviation. The sensors were calibrated using a third degree polynomial.

The achieved Root Mean Square Error (RMSE) of the compression sensor was 0.24 ± 0.2N and 0.4 ± 0.29N for sensor used to measure the tension. Although identical with respect to model and manufacturer, the two sensors showed a substantial difference in accuracy, with the sensor used for tension producing a larger hysteresis error in particular. It can therefore be concluded that an initial characterization of multiple sensors is advisable, in order to select the best performing specimen for the system.

### 5.2 ROM Evaluation of the Exoskeleton

**FIGURE 12:**
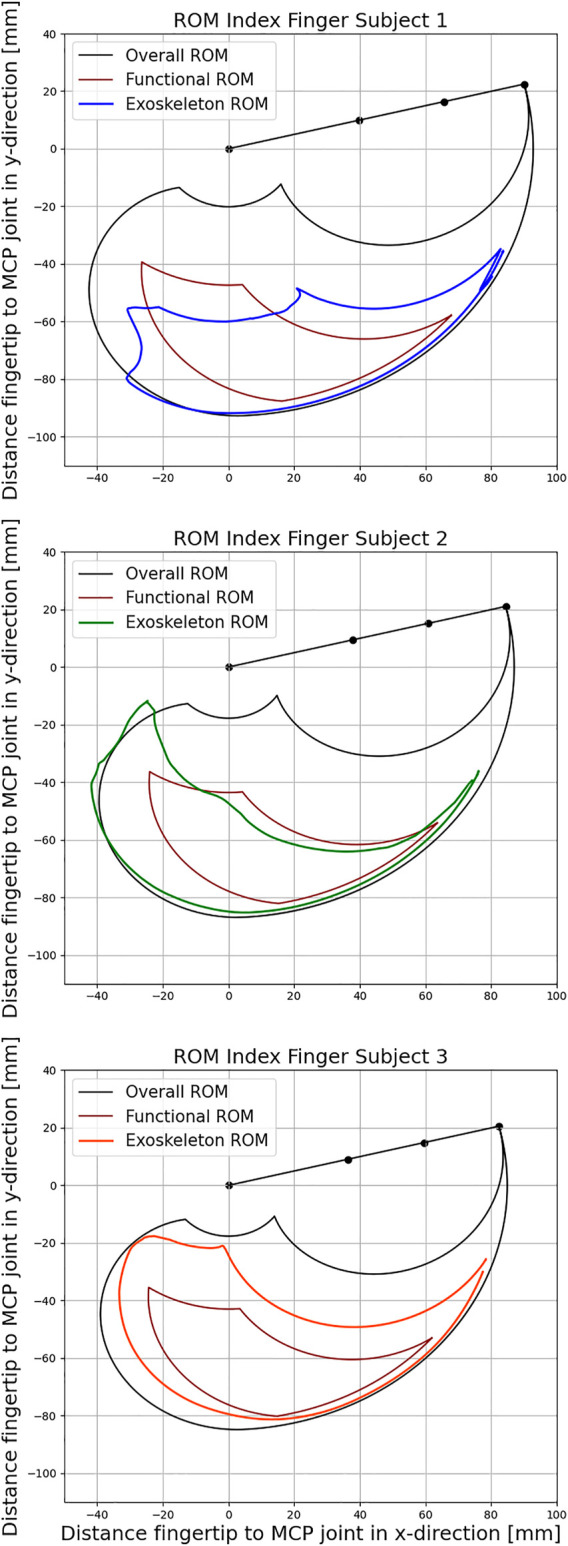
Comparison of the exoskeleton Range of Motion (ROM) with active and functional movement space for the three subjects respectively according to Bain et al. (2015).

Since the exoskeleton is intended to be used for rehabilitation purposes, the ROM covered by the system is a critical factor. Figure 12 shows the maximum achievable ROM of the exoskeleton compared to the active and functional ROM of a finger with the same phalanx length using data collected by Bain et al. (2015) for each subject. For this experiment, each subject was instructed to perform a repetitive motion with maximum flexion and extension of the index finger using the exoskeleton as a measurement tool, and the corresponding joint trajectories were determined by model evaluation.

The active ROM (black curve) represents all positions that the fingertip can potentially reach. The functional ROM (red curve), on the other hand, represents a subset of these positions that are needed to perform the majority of tasks in everyday life. The ROM achieved by the coupling of the Exoskeleton with the index finger of the subjects are represented by the blue, green and orange curve.

The individual data of the subjects as well as the resulting Range of Motion (ROM) are listed in Table 1. Evaluation of the functional ROM reveals that the coupling between the exoskeleton and the index finger includes 77, 87, and 100*%*, respectively, for the three subjects. As the length of the index finger decreases from 93 to 87 mm and 85 mm, the corresponding functional ROM increases as the kinematic structure becomes relatively larger and can travel further distances during flexion. Positions that require strong flexion of all three finger joints are therefore not achievable for subject 1. At the same time, all exoskeleton couplings are able to provide almost unrestricted movements in the lower right part of the functional ROM. Therefore, the exoskeleton is well suited for training a variety of different grasping tasks from daily life for all subjects.

**TABLE 1:** Tabulated parameters of the three individual subjects for the index finger and the achievable Range of Motion (ROM).

	Subject 1	Subject 2	Subject 3
Gender	male	female	female
Age (years)	27	25	25
Overall Length (mm)	93	87	85
Proximal Phalanx (mm)	41	39	37
Middle Phalanx (mm)	27	24	24
Distal Phalanx (mm)	25	24	24
Functional ROM	77*%*	87*%*	100*%*
Overall ROM	40*%*	44*%*	60*%*

### 5.3 Angle and Torque Measurement

**FIGURE 13:**
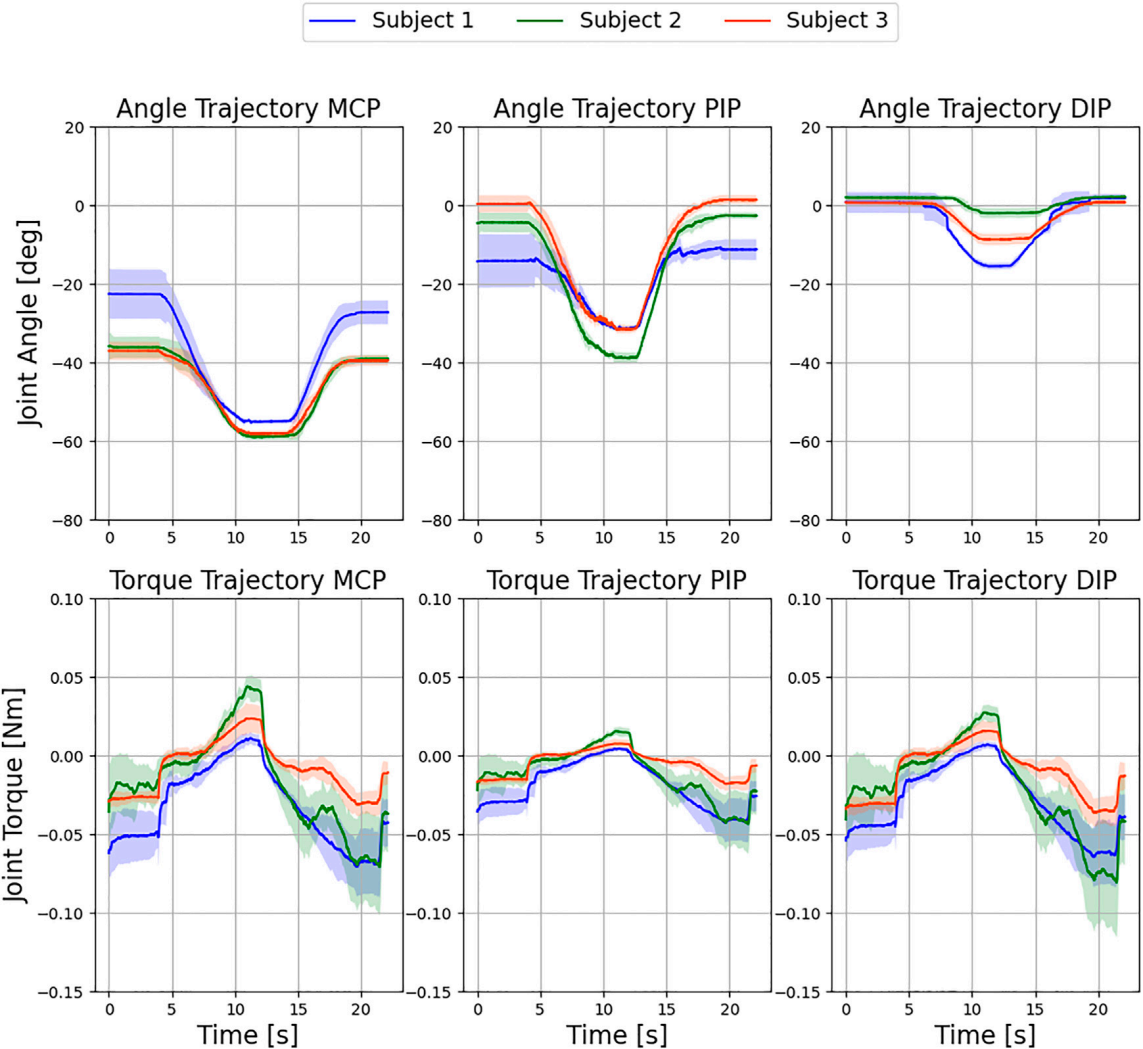
Evaluation of exemplary movement of the three fingers with the exoskeleton over six independent test cycles. Displayed are the resulting mean ± standard deviation over all cycles for the respective subjects. No control intervention occurred.

The purpose of this series of experiments was to further demonstrate the exoskeleton’s ability to capture the angular and torque trajectory of each finger joint using the presented sensor control system. To replicate the trajectories, raw data from the sensors was recorded and analyzed in Python. Figure 13 shows the results of the study conducted with the three subjects. All three finger joints were flexed and extended by the exoskeleton in a natural motion. Specifically, the experiment was conducted in free air with no obstacles to the finger trajectories, and the subjects were instructed to relax their hand during the experiment. The actuator followed a sinusoidal linear motion. For the interpretation of the sensor data, the kinematic configuration was recreated for each record of the recorded angles. Then, the forces acting on the finger were calculated using the force equilibrium model, and finally, the quasi-static joint moments were computed using the simplified dynamic model. The obtained trajectories consequently provide conclusions about the individual joint stiffness and damping of the subjects.

As can be seen in Figure 13, the standard deviation of the angular trajectories around the point of maximum flexion decreases for all finger joints across all subjects. One reason for this is that the adaptive system allows the finger more freedom in the straight position. With increasing flexion, the index finger follows its natural trajectory for all subjects and the deviation between the measurements decreases. In addition, the low standard deviation is direct evidence of a reproducible setup with sufficient finger fixation and satisfactory exoskeleton stiffness. The moment trajectories between the MCP and DIP joints are very similar in all subjects and thus carry a comparatively large portion of the external load. In comparison, the PIP joint shows a flatter trajectory and is loaded less. This can be better explained by looking at the angular trajectories. While the MCP and DIP joints reach a plateau at maximum flexion, the PIP joint continues to rotate until the reversal point and is thus hardly loaded at all.

### 5.4 Force Control Interaction

**FIGURE 14:**
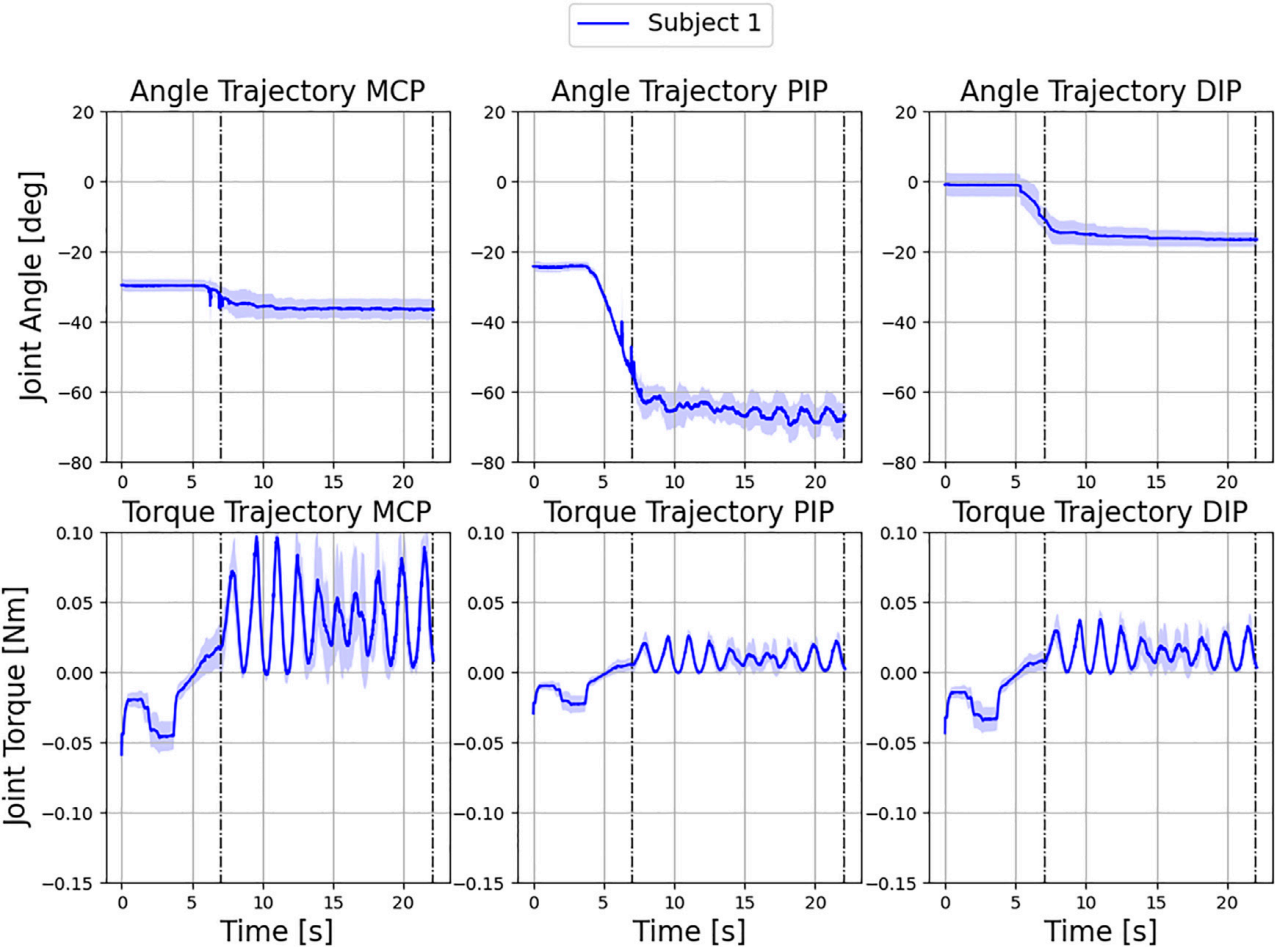
Control intervention for subject 1 during a flexing movement of the index finger with a torque limit of 0.1 Nm at the actuator over three independent test cycles. Displayed are the resulting mean ± standard deviation over all cycles. The finger hits a force sensitive plate at 7 s, which measures a maximum external force to the fingertip of 1.53 ± 0.1 N.

Finally, the force control system was tested by restricting the movement of the exoskeleton in subject 1. This involved installing a force-sensitive plate as an obstacle to the free movement of the finger and measuring the resulting force at the fingertip. To characterize the force transfer, the driving torque on the exoskeleton was limited to 0.1 Nm. Since the dynamic model does not account for the external contact force acting on the finger, the resistive forces acting on the fingertip are interpreted by the model as increased joint stiffness. Figure 14 shows a steep increase in joint moments after the finger hits the force sensitive plate in the figure (indicated by the first dashed line).

In the area of contact with the plate, an oscillation induced by the controller can be observed, which can be explained by the maximum and minimum permissible torques of the drive torque. As soon as the threshold value (0.1 Nm) of the two-point controller is reached (0.01 Nm), the control direction reverses. The corresponding maximum external force on the force sensitive plate is 1.53 ± 0.1 N. As a direct consequence, the measured torques drop immediately. As soon as the lower threshold value of the controller is reached, the original direction is restored. This effect shows up differently in the torque curves of the individual finger joints. While the DIP and PIP joints are loaded with rather low torques due to the action of the external contact force, the torque in the MCP joint accumulates more intensively, which can be explained by a larger lever arm of the external force. In the angular trajectories, the MCP and DIP joints remain almost unchanged. Only the PIP joint reacts to the changed load and executes the motion induced by the changed driving force. The overall low standard deviation of the trajectories can be taken as evidence of sufficient load-bearing capacity of the system in subject 1. No drift was observed during the measurements, as the finger attachments remained in place and the induced force application to the surface was reproducible. As this experiment shows, the intensity of the exercises can be adapted to the functional state of the patient’s hand by adjusting the upper and lower thresholds of the controller, so that the therapy is always comfortable for the patient. In summary, the experiments performed demonstrate the ability of the exoskeleton to cover a satisfactorily large functional ROM (77, 87, and 100%), the possibility to obtain accurate patient data that can be used for clinical diagnosis and for the quantification of rehabilitation success over time. The experiments also show the capability of the system to be used in rehabilitation due to the force-limiting control algorithm.

## 6 Conclusion

In this paper, a force-controlled exoskeleton for finger rehabilitation was presented. The development goal was to provide a system suitable for automated rehabilitation of CRPS patients. The presented exoskeleton satisfies essential requirements for the treatment of CRPS patients. Therefore, its application can reduce the cost of treatment while enabling a higher frequency of rehabilitation exercises. Through the integrated position and force sensors, the system collects data to objectively quantify the functional status of the finger. This data includes the finger’s ROM, as well as angular and torque trajectories of each finger joint, allowing the therapist to track rehabilitation progress and evaluate treatment effectiveness. In particular, the recorded resistive torque exhibited by each joint at different angular positions during finger movement can be used to assess changes in joint stiffness over time. Due to the adaptive nature of the exoskeleton’s kinematic structure, a variety of different force-assisted grasping tasks can be performed with the exoskeleton. The actuation force acting on the kinematic structure is measured and limited by a two-point controller, which in turn limits the maximum possible torque at each finger joint. In the current configuration, the exoskeleton can provide passive support by applying force to the fingertip in both directions. The functional ROM covered by the exoskeleton is satisfactory for rehabilitation purposes. The system is easy to attach and detach from the patient’s finger and is suitable for use in the home due to its low complexity and cost.

### 6.1 Future Work

In the future, the kinematic model can be extended in order to allow analytical calculation of the finger joint velocities and accelerations. This will allow to fully leverage the potential of the created dynamic finger model. Further, it will enable doctors to differentiate between dry friction and viscous friction in the finger joints based on the recorded patient data. The system can also be extended to cover the remaining fingers of the hand. The control algorithm will be extended to allow active and active–assistive modes in addition to passive rehabilitation, in which the exoskeleton allows patients to move their hands freely and only applies assistive forces when patients cannot perform a specific movement through their own finger strength. In addition, a prototype will be developed for clinical testing. For this purpose, a dorsal support for the exoskeleton will be designed that allows the kinematic structure for each finger to follow the movement of the back of the hand during exercises such as spherical grasping.

## Data Availability

The datasets presented in this study can be found in online repositories. The names of the repository/repositories and accession number(s) can be found below: https://github.com/NikonPic/ExoEval.

## References

[B1] Aragón-Martínez A. Arias-Montiel M. Lugo-González E. Tapia-Herrera R. (2020). Two-finger Exoskeleton with Force Feedback for a mobile Robot Teleoperation. Int. J. Adv. Robotic Syst. 17, 172988141989564. 10.1177/1729881419895648

[B2] Bain G. I. Polites N. Higgs B. G. Heptinstall R. J. McGrath A. M. (2015). The Functional Range of Motion of the finger Joints. The J. Hand Surg. Eur. volume 40, 406–411. 10.1177/1753193414533754 24859993

[B3] Birch B. Haslam E. Heerah I. Dechev N. Park E. J. (2008). Design of a Continuous Passive and Active Motion Device for Hand Rehabilitation. IEEE Eng. Med. Biol. Soc. Conf. 2008, 4306–4309. 10.1109/IEMBS.2008.4650162 19163665

[B4] Carbone G. Gerding E. C. Corves B. Cafolla D. Russo M. Ceccarelli M. (2020). Design of a Two-DOFs Driving Mechanism for a Motion-Assisted finger Exoskeleton. Appl. Sci. 10, 2619. 10.3390/app10072619

[B5] Cempini M. Cortese M. Vitiello N. (2015). A Powered finger–thumb Wearable Hand Exoskeleton with Self-Aligning Joint Axes. IEEE/ASME Trans. Mechatronics 20, 705–716. 10.1109/TMECH.2014.2315528

[B6] Chiri A. Giovacchini F. Vitiello N. Cattin E. Roccella S. Vecchi F. (2009). “Handexos: Towards an Exoskeleton Device for the Rehabilitation of the Hand,” in 2009 IEEE/RSJ International Conference on Intelligent Robots and Systems, October 11-15, 2009, St. Louis, MO, IEEE, 1106–1111. 10.1109/IROS.2009.5354376

[B7] Conti R. Meli E. Ridolfi A. Bianchi M. Governi L. Volpe Y. (2017). Kinematic Synthesis and Testing of a New Portable Hand Exoskeleton. Meccanica 52, 2873–2897. 10.1007/s11012-016-0602-0

[B8] Di Guardo A. Sarac M. Gabardi M. Leonardis D. Solazzi M. Frisoli A. (2019). “Sensitivity Analysis and Identification of Human Parameters for an Adaptive, Underactuated Hand Exoskeleton,” in Advances in Robot Kinematics 2018. Editors Lenarcic J. Parenti-Castelli V. Cham: Springer International Publishing, 449–457.

[B9] Elsamadicy A. A. Yang S. Sergesketter A. R. Ashraf B. Charalambous L. Kemeny H. (2017). Prevalence and Cost Analysis of Complex Regional Pain Syndrome (CRPS): A Role for Neuromodulation. Neuromodulation: Techn. Neural Interf. 21, 423–430. 10.1111/ner.12691 PMC5876058 28961359

[B10] Epstein D. Mason A. Manca A. (2008). The Hospital Costs of Care for Stroke in Nine European Countries. Health Econ. 17, S21–S31. 10.1002/hec.1329 18186037

[B11] Festo AG and Co. KG (2012). Exohand.

[B12] Heo P. Gu G. M. Lee S.-j. Rhee K. Kim J. (2012). Current Hand Exoskeleton Technologies for Rehabilitation and Assistive Engineering. Int. J. Precision Eng. Manufacturing 13, 807–824. 10.1007/s12541-012-0107-2

[B13] Iqbal J. Khan H. Tsagarakis N. G. Caldwell D. G. (2014). A Novel Exoskeleton Robotic System for Hand Rehabilitation – Conceptualization to Prototyping. Biocybernetics Biomed. Eng. 34, 79–89. 10.1016/j.bbe.2014.01.003

[B14] Iqbal J. Khelifa B. (2014). Stroke Rehabilitation Using Exoskeleton-Based Robotic Exercisers: Mini Review. Biomed. Res. 26, 197–201.

[B15] Iqbal J. Tsagarakis N. Fiorilla A. Caldwell D. (2009). “Design Requirements of a Hand Exoskeleton Robotic Device,” in Proceedings of the 14th IASTED International Conference on Robotics and Applications (RA 2009), November 2-4, 2009, Cambridge, MA, 44–51.

[B16] Jo I. Bae J. (2017). Design and Control of a Wearable and Force-Controllable Hand Exoskeleton System. Mechatronics 41, 90–101. 10.1016/j.mechatronics.2016.12.001

[B17] Kerle H. Corves B. Hüsing M. (2015). Getriebetechnik (Wiesbaden. Springer Fachmedien Wiesbaden. 10.1007/978-3-658-10057-5

[B18] Lee J. Lee M. Bae J. (2018). Development of a Hand Exoskeleton System for Quantitative Analysis of Hand Functions. J. Bionic Eng. 15, 783–794. 10.1007/s42235-018-0066-0

[B19] Leonardis D. Barsotti M. Loconsole C. Solazzi M. Troncossi M. Mazzotti C. (2015). An Emg-Controlled Robotic Hand Exoskeleton for Bilateral Rehabilitation. IEEE Trans. Haptics 8, 140–151. 10.1109/TOH.2015.2417570 25838528

[B20] Maihöfner C. Seifert F. Markovic K. (2010). Complex Regional Pain Syndromes: New Pathophysiological Concepts and Therapies. Eur. J. Neurol. 17, 649–660. 10.1111/j.1468-1331.2010.02947.x 20180838

[B21] Mark V. W. Taub E. (2004). Constraint-induced Movement Therapy for Chronic Stroke Hemiparesis and Other Disabilities. Restorative Neurol. Neurosci. 22, 317–336. 15502259

[B22] McCarthy J. M. Soh G. S. (2011). Geometric Design of Linkages, 11. New York, NY: Springer New York. 10.1007/978-1-4419-7892-9

[B23] Patton J. L. Mussa-Ivaldi F. A. (2004). Robot-assisted Adaptive Training: Custom Force fields for Teaching Movement Patterns. IEEE Trans. bio-medical Eng. 51, 636–646. 10.1109/TBME.2003.821035 15072218

[B24] Reinkensmeyer D. J. Emken J. L. Cramer S. C. (2004). Robotics, Motor Learning, and Neurologic Recovery. Annu. Rev. Biomed. Eng. 6, 497–525. 10.1146/annurev.bioeng.6.040803.140223 15255778

[B25] Sarac M. Solazzi M. Sotgiu E. Bergamasco M. Frisoli A. (2016). Design and Kinematic Optimization of a Novel Underactuated Robotic Hand Exoskeleton. Meccanica 52, 749–761. 10.1007/s11012-016-0530-z

[B26] Taub E. Miller N. E. Novack T. A. Cook E. W. Fleming W. C. Nepomuceno C. S. (1993). Technique to Improve Chronic Motor Deficit after Stroke. Arch. Phys. Med. Rehabil. 74, 347–354. 8466415

[B27] Tyromotion (2019). Amadeo - Tyromotion.

[B28] Wang J. Li J. Zhang Y. Wang S. (2009). “Design of an Exoskeleton for index finger Rehabilitation,” in Conference proceedings : 31st Annual International Conference of the IEEE Engineering in Medicine and Biology Society, Setember 3-6, 2009, Minneapolis, MN, IEEE Engineering in Medicine and Biology Society. 10.1109/IEMBS.2009.5334779 19965067

[B29] Wege A. Kondak K. Hommel G. (2006). “Development and Control of a Hand Exoskeleton for Rehabilitation,” in Human Interaction with Machines. Editors Hommel G. Huanye S. Dordrecht: Springer Netherlands, 149–157.

[B30] Yue Z. Zhang X. Wang J. (2017). Hand Rehabilitation Robotics on Poststroke Motor Recovery. Behav. Neurol. 2017. 10.1155/2017/3908135 PMC5688261 29230081

